# Diagnosis of rifampicin-resistant tuberculosis: Discordant results by diagnostic methods

**DOI:** 10.4102/ajlm.v7i2.806

**Published:** 2018-12-06

**Authors:** Winnie Mwanza, Deborah Milimo, Maureen M. Chilufya, Nkatya Kasese, Maina C. Lengwe, Stembiso Munkondya, Petra de Haas, Helen Ayles, Monde Muyoyeta

**Affiliations:** 1 Zambia AIDS Related Tuberculosis (ZAMBART) Project, School of Medicine, University of Zambia, Lusaka, Zambia; 2 Department of Infectious and Tropical diseases, London School of Hygiene and Tropical Medicine, Bloomsburg, London, United Kingdom

## Abstract

The performance of the Xpert© MTB/RIF and MTBDR*plus* assays for the detection of rifampicin resistant *Mycobacterium tuberculosis* was compared to culture-based drug susceptibility testing in 30 specimens with rifampicin-resistant and rifampicin-indeterminate Xpert MTB/RIF results collected between March 2012 and March 2014. Xpert MTB/RIF and MTBDR*plus* were 100% sensitive and 100% concordant for rifampicin resistance detection, but 3 of 13 samples (23%) positive for rifampicin resistance on Xpert MTB/RIF and MTBDR*plus* were negative for rifampicin resistance on mycobacteria growth indicator tube drug susceptibility testing. Specificity was 72% for Xpert MTB/RIF and 80% for MTBDR*plus*. Positive predictive value for Xpert MTB/RIF for multidrug resistant tuberculosis was 47.8% for new patients and 77.8% for previously treated patients; negative predictive value was 100% for both new and previously treated patients. The discordant rifampicin resistance test results indicate a need to fully characterise circulating rifampicin resistant *Mycobacterium tuberculosis* strains in Zambia and to inform the development of guidelines for decision-making in relation to diagnosis of drug-resistant tuberculosis.

## Introduction

Management and control of multidrug resistant (MDR) tuberculosis, tuberculosis that is resistant to both isoniazid and rifampicin, relies on timely and correct diagnosis. The World Health Organization has endorsed the Xpert®MTB/RIF (Cepheid Sunnyvale, CA, United States) and GenoType MTBDR*plus* (HAIN Life Sciences, Nehren, Germany) tests for rapid diagnosis of MDR tuberculosis.^1,2^ For the Xpert MTB/RIF test, the current recommendation is to treat any rifampicin-resistant individual as a case of MDR tuberculosis until proven otherwise by subsequent drug susceptibility testing, after which treatment is to be tailored or individualised accordingly. The combination of Xpert MTB/RIF and MTBDR*plus* test results would be a powerful tool to achieve prompt confirmation of diagnosis of MDR tuberculosis. These genotypic methods, however, do not detect resistance conferred by mutations outside of the targeted gene region.^3,4,5^

We report comparative observations made in a pilot study on the performance of Xpert MTB/RIF, MTBDR*plus* and Mycobacteria Growth Indicator Tube (MGIT) drug susceptibility testing methods for detection of rifampicin resistance in Lusaka, Zambia, at the Zambart Central Laboratory (ZCL).

## Methods

### Ethical considerations

The study was approved by the University of Zambia biomedical research ethics committee (ref. no. 003-12-11) and all patients were requested to give written informed consent before participating in the study. The written informed consent was administered by study staff, and participants were given an opportunity to discuss the study before signing the informed consent form. To maintain confidentiality, each participant was allocated a unique study number that was used on all data collection tools, as well as for labelling of laboratory specimens. No names were used on any data collection tool. Informed consent forms and all study data were kept in secure cabinets with access limited to the principal investigator and the data manager. Paper-based case report forms were captured into secured, password-protected electronic databases.

### Study Design

The study was conducted between March 2012 and March 2014 on samples collected during an evaluation of Xpert MTB/RIF use in primary health care facilities in Lusaka, Zambia.^6,7^ Samples were collected from consecutive patients presenting to the primary health care facility who had rifampicin-resistant and rifampicin-indeterminate Xpert MTB/RIF results for confirmation of rifampicin resistance and for quality assurance in the ZCL.

Samples were transported to the central laboratory on the same day of collection or the following day and no preservatives were added. At the ZCL, the samples were decontaminated using the NaOH/NaLC method (2% NaOH for 20 minutes) and 0.5 mL of the pellet was inoculated into each of two manual MGIT tubes for primary isolation and identification of *M. tuberculosis*.; 0.5 mL was used to repeat the Xpert MTB/RIF test, and two drops were used to prepare a concentrated smear for fluorescent microscopy. 1.5 mL of the Xpert MTB/RIF sample reagent was added to 0.5 mL of the re-suspended pellet and Xpert MTB/RIF testing was performed as per standard procedure described by the manufacture.

Acid-fast bacilli positive cultures were identified using the capillia tuberculosis Neo assay (TAUNS Laboratories, Inc., Shizuoka, Japan) to confirm presence of *Mycobacterium tuberculosis* complex.^8^ Confirmed *M. tuberculosis* complex isolates were tested for drug sensitivity using the Becton Dickinson manual MGIT SIRE (streptomycin, isoniazid, rifampicin, ethambutol; Becton Dickinson, Bergen County, New Jersey, United States) drug susceptibility testing method and the MTBDR*plus* assay (version 1; HAIN Life Sciences, Nehren, Germany). The drug concentrations used were 1.0 µg/mL streptomycin, 0.1 µg/mL isoniazid, 1.0 µg/mL rifampicin, and 5.0 µg/mL ethambutol. The MGIT SIRE tubes were read daily as per the manufacturer’s recommendation. To perform the MTBDR*plus* assay, 1 mL of broth from an *M. tuberculosis* complex culture-positive MGIT tube was used for DNA extraction using the genolyse method. Briefly, 1 ml of culture broth was centrifuged, the pellet was incubated for 5 minutes at 95°C in lysis buffer after which a neutralisation buffer was added. Subsequent steps for polymerase chain reaction and hybridisation were followed as per standard procedure for MTBDR*plus* testing.^9^ All procedures included positive and negative controls.

The data were analysed using Stata version 13 (StataCorp LLC, College Station, Texas, United States). An initial descriptive analysis was done to determine the distribution of results, then the Xpert MTB/RIF results obtained at the primary health care setting were compared with the results obtained at the central laboratory. Further analysis was done to compare the performance of Xpert MTB/RIF, MTBDR*plus* and MGIT *M. tuberculosis* drug susceptibility testing and to determine the sensitivity and specificity of Xpert MTB/RIF and MTBDR*plus* compared to the gold standard MGIT culture for drug susceptibility testing.

## Results

During the study period, 1070 patients had *M. tuberculosis* complex detected by the Xpert MTB/RIF test. Of these, 28 (2.6%) were positive for rifampicin resistance and 15 (1.4%) had indeterminate rifampicin resistance results. Of the 43 patients eligible for inclusion in the study (i.e. had rifampicin-resistant or rifampicin-indeterminate Xpert MTB/RIF results), only 30 submitted samples for further testing at the central laboratory (Table 1). Of these, 21 (70%) were positive for both *M. tuberculosis* and rifampicin resistance, and nine (30%) were positive for *M. tuberculosis* but indeterminate for rifampicin resistance. On repeat Xpert MTB/RIF testing at the central laboratory, of the 21 patients initially positive for rifampicin resistance, 17 (81%) were again positive for rifampicin resistance, three (14%) were negative for rifampicin resistance and one (5%) tested negative for *M. tuberculosis*.

**TABLE 1:** Comparison of Xpert MTB/RIF testing at the primary health care facility peripheral laboratory to Xpert MTB/RIF testing at the central laboratory, Zambia, March 2012 and March 2014.

Primary laboratory Xpert MTB/RIF test result	Number	Central laboratory repeat Xpert MTB/RIF test result		
		Negative for *M. tuberculosis*	Not rifampicin resistant	Rifampicin resistant
Indeterminate	9	4 (44.4%)	5 (55.6%)	0
Rifampicin resistant	21	1 (4.8%)	3 (14.3%)	17 (81.0%)

**Total**	**30**	**5 (16.7%)**	**8 (26.7%)**	**17 (56.6%)**

Of the nine patients initially indeterminate for rifampicin resistance, five (56%) tested negative for rifampicin resistance and four (44%) tested negative for *M. tuberculosis* on repeat Xpert MTB/RIF testing (Table 1). These four (44%) samples negative for *M. tuberculosis* on repeat Xpert MTB/RIF testing were *M. tuberculosis* positive but rifampicin resistance negative when tested by both MTBDRplus and MGIT drug susceptibility testing (Tables 2–3). Xpert MTB/RIF and MTBDR*plus* were 100% concordant for detection of rifampicin resistance (Table 2). When compared to MGIT drug susceptibility testing, the sensitivity of Xpert MTB/RIF and MTBDR*plus* for detection of rifampicin resistance was 100%, while specificity was 72% for Xpert MTB/RIF and 80% for MTBDR*plus*. Three patients had positive rifampicin resistance results on both Xpert MTB/RIF and MTBDR*plus* (Table 2), but rifampicin resistance was not detected with MGIT drug susceptibility testing (Tables 3 and 4). On Xpert MTB/RIF all three samples showed no signal for probe D and on MTBDR*plus*, all were missing the wild-type 7 probe and showed no mutation probe (results not shown). These three isolates were also resistant to isoniazid on both MGIT drug susceptibility testing and MTBDR*plus* (Tables 3 and 4).

**TABLE 2:** Comparison of repeat Xpert MTB/RIF and MTBDR*plus* test results.

Repeat Xpert MTB/RIF test result	Number	MTBDR*plus*				
		Sensitive to isoniazid and rifampicin	Multidrug resistant	Isoniazid resistant	Rifampicin resistant	No result
Rifampicin resistant	17	0	13	0	3	1
Not rifampicin resistant	8	8	0	0	0	0
Negative for *M. tuberculosis*	5	2	1	2	0	0

**Total**	**30**	**10**	**14**	**2**	**3**	**1**

**TABLE 3:** Comparison of repeat Xpert MTB/RIF and mycobacterium growth indicator tube *Mycobacterium tuberculosis* drug susceptibility testing.

Repeat Xpert MTB/RIF test result	Number	MGIT *M. tuberculosis* drug susceptibility testing				
		Sensitive to isoniazid and rifampicin	Multidrug resistant	Isoniazid resistant	Rifampicin resistant	No result
Rifampicin resistant	17	0	10	3	3	1
Not rifampicin resistant	8	8	0	0	0	0
Negative for *M. tuberculosis*	5	2	1	2	0	0

**Total**	**30**	**10**	**11**	**5**	**3**	**1**

Abbreviations: MGIT, mycobacterium growth indicator tube.

**TABLE 4:** Comparison of MTBDR*plus* and mycobacterium growth indicator tube *Mycobacterium tuberculosis* drug susceptibility testing.

MTBDR*plus* result	Number	MGIT *M. tuberculosis* drug susceptibility testing				
		Sensitive to isoniazid and rifampicin	Multidrug resistant	Isoniazid resistant	Rifampicin resistant	No result
Sensitive to isoniazid and rifampicin	10	10	0	0	0	0
Multidrug resistant	14	0	11	3	0	0
Isoniazid mono resistant	2	0	0	2	0	0
Rifampicin mono resistant	3	0	0	0	3	0
No result	1	0	0	0	0	1

**Total**	**30**	**10**	**11**	**5**	**3**	**1**

Abbreviations: MGIT, mycobacterium growth indicator tube.

The overall positive predictive values and negative predictive values for MDR tuberculosis diagnosis for Xpert MTB/RIF compared to MGIT drug susceptibility testing were 62.5% and 100% and compared to MTBDR*plus* were 81.2% and 100% (results not shown). For previously treated patients, the Xpert MTB/RIF positive predictive value was 77.8% and negative predictive value was 100%; for new patients, positive predictive value was 47.8% and negative predictive value was 100%. The MTBDR*plus* positive predictive value was 78.6% and negative predictive value was 100% compared to MGIT.

## Discussion

Indeterminate rifampicin resistance results can be resolved by repeating the test, as shown in our study. Of all the samples that were initially indeterminate for rifampicin resistance, none tested positive on repeat testing. An indeterminate rifampicin resistance Xpert MTB/RIF result maybe due to low DNA quantity in the sample; this was supported by the high proportion of samples that were initially indeterminate that did not detect *M. tuberculosis* on repeat testing with Xpert MTB/RIF but were positive on *M. tuberculosis* culture. For prediction of MDR tuberculosis, our findings confirm that Xpert MTB/RIF has a low positive predictive value for MDR tuberculosis in low-prevalence MDR tuberculosis settings like Zambia, which has an estimated MDR prevalence of 4.2%.^10^ Depending on Xpert MTB/RIF alone for diagnosis of MDR tuberculosis in our setting would lead to subjecting a significant proportion of patients to unnecessary second-line treatment.^1^

The performance of Xpert MTB/RIF and MTBDR*plus* were similar, which was expected since both are genotypic tests. The sensitivity of Xpert MTB/RIF for detection of rifampicin resistance was within the range of what has been reported by others, whereas the specificity of 73% was lower.^11^ The prevalence of silent *rpoB* gene mutations that do not confer phenotypic resistance determines the specificity of genotypic tests, if using a phenotypic test as a gold standard. While we were limited by a lack of capacity to sequence the three samples that had results suggesting false rifampicin resistance, other studies have reported false Xpert MTB/RIF or MTBDR*plus* rifampicin resistance caused by silent mutations in the *rpoB* gene.^12,13,14,15,16^ Further, these three isolates were also resistant to isoniazid on both MGIT drug susceptibility testing and MTBDR*plus*, consistent with the widely observed fact that in most cases, silent mutations in the *rpoB* gene are associated with mutations in the *katG* gene or promoter of the *inhA* gene.^17,18^ Probe D in the Xpert MTB/RIF and the wild-type 7 probe in MTBDR*plus* both target the same region of the *rpoB* gene, codon 526, which is one of the regions with known mutations that confer resistance to rifampicin. Detection of mutations in this region by both Xpert MTB/RIF and MTBDR*plus* shows strong evidence of the accuracy of our genotypic results. However, MGIT drug susceptibility testing has been shown to produce false susceptibility results for strains with minimal inhibitory concentrations for rifampicin close to the cut-off value of 1 µg/mL used in MGIT drug susceptibility testing, or strains that have sub-critical minimal inhibitory concentrations for rifampicin.^19^ However, it is unlikely that the discordant results in our study were due to false susceptibility results for the MGIT drug susceptibility testing. To resolve these discrepant results, sequencing is required to determine the presence of silent mutations. However, lack of access to sequencing services was a limitation in our study.

This study was also limited by its small sample size but provides some insights into the comparable performance of Xpert MTB/RIF, MTBDR*plus* and culture drug susceptibility testing culture for the diagnosis of drug-resistant tuberculosis. There is an urgent need for Zambia to perform a full identification and classification of *rpoB* mutations and investigate minimal inhibitory concentrations for rifampicin, so as to optimise national guidelines for diagnosis of drug-resistant tuberculosis. A thorough investigation of the performance of Xpert MTB/RIF and MTBDR*plus* for diagnosis of rifampicin resistance and prediction of MDR tuberculosis in the Zambian setting is recommended to avoid inappropriate treatment. Whole genome sequencing capacity is required to fully characterise circulating rifampicin-resistant *M. tuberculosis* strains. Improvements are needed to make these genotypic tests function as stand-alone tests, as they offer the best prospects for early and accurate diagnosis for tuberculosis and MDR tuberculosis.
